# Do genomic passports leave us more vulnerable or less vulnerable? Perspectives from an online citizen engagement

**DOI:** 10.1057/s41599-023-01580-7

**Published:** 2023-03-04

**Authors:** Chloé Mayeur, Heidi Mertes, Wannes Van Hoof

**Affiliations:** 1 Sciensano, Ixelles, Belgium; 2 Ghent University, Ghent, Belgium

**Keywords:** Ethics, Science, technology and society

## Abstract

Since genomics is becoming commonplace in healthcare for the diagnosis, treatment, and prevention, the prospect of generating a genomic passport for all citizens is gaining traction. While this would have many advantages, it raises ethical issues requiring societal debate alongside academic reflection. Hence, Sciensano—the Belgian scientific Institute of Public Health—organised an online citizen engagement on genomic information usage, including a question on a genomic passport for all. The inductive thematic analysis of participants’ contributions highlighted vulnerability as a fundamental concern, while this has not received sufficient attention so far in genomics. Participants expressed their vulnerability in two ways. First, the genomic passport would inform them about their ontological vulnerability. By revealing their constitutional weaknesses (predisposition to diseases), it reminds them that everyone is unavoidably and perennially at risk of being harmed. Second, the misuse of the genomic passport can add situational vulnerabilities (e.g., discrimination causing psychological and economic harm). Moreover, the fundamental uncertainty in genomics—how will such sensitive information be used, and how will the science evolve?—exacerbates these vulnerabilities. This article ends with recommendations to alleviate these vulnerabilities in genomics now and in the future in which the genomic passport may become a reality.

## Introduction

As the use of genomic technologies is becoming commonplace in healthcare for diagnostics and prevention, the prospect of sequencing the genomes of citizens at a large scale and sharing those genomic data to improve public health and for research aimed at enabling personalised healthcare is gaining traction. For this article, we will call this concept the genomic passport.

The concept of a genomic passport has already been studied in some specific contexts. In 2019, researchers developed the PGx-Passport, a panel of 58 actionable germline variants alleles for pharmacogenetic purposes (van der Wouden et al., 2019). In 2022, Gyngell and Savulescu proposed that a genomic passport could help manage COVID-19 and future pandemics (Gyngell and Savulescu, 2022). Additionally, the European Society of Human Genetics argued that the broad implementation of whole-genome sequencing could lead to faster diagnosis of genetic diseases, personalised treatments, and improved public health (van El et al., 2013). Lastly, Genomics England organised a public engagement initiative on whole-genome sequencing in newborns within the context of an early-stage pilot study. They aimed to examine whether it could replace or be an alternative to the current newborn screening, whose limited purposes would be extended to pharmacogenomics, lifetime monitoring, research, and family planning (Hopkins et al., 2021). The genomic passport is thus not a fantasy but a tangible future whose ethical and societal implications need to be discussed now. In this way, its future implementation could be guided by ethics instead of a technological imperative that may neglect critical ethical considerations.

Aside from academic ethical reflection, societal debate and public engagement can offer relevant contributions to the ethics of genomic passports. Engaging the public on this topic is critical for several reasons:

- Genomics impacts, or will impact, citizens’ lives in many different and private ways. Public engagement ensures that genomics grows within a widely supported framework that considers citizens’ concerns and values rather than blindly following the technological imperative. It can be a tool to empower different audiences regarding how their genomic information is used.
- Experts and policymakers need the input of citizens to understand public concerns about genomics and find solutions for them. For instance, several reasons may explain the reluctant uptake of genomics despite the potential benefits.
- It may improve citizens’ trust and make them valuable partners in genomics.

Several professional associations, experts in genomics, and public health institutes have already pointed out the need for public engagement in standard or population-based genetic screenings (Hopkins et al., 2021; van El et al., 2013; Grosse et al., 2010; Tarini et al., 2010; Moyer et al., 2008). In 2019–2020, Sciensano—the Scientific Institute of Public Health in Belgium—organised the online DNA debate, a broad and open public engagement initiative on genomic information usage that included one specific question on the eventuality of a genomic passport for all citizens. Aside from raising public awareness, this initiative aimed at producing and presenting recommendations based on citizens’ perspectives to health policymakers, experts, and stakeholders in genomics.

This article highlights the values, concerns, and motivations that participants of the DNA debate put forward when confronted with the idea of a genomic passport for all. However, most importantly, it focuses on vulnerability as a central concern that anyone using genomic data should bear in mind.

## Methods

There is a long history of applying methods from public deliberation to health-related topics, particularly concerning complex issues and ethics (Abelson et al., 2013). Over the years, these methods have evolved into the digital space where online participation offers new options to researchers and policymakers (Friess and Eilders, 2015; Monnoyer-Smith and Wojcik, 2012; Davies and Gangadharan, 2009). Especially the combination of online and offline engagement activities increases the variety of discourses and opens the scope of the discussions (Monnoyer-Smith and Wojcik, 2012). The Belgian DNA debate was an online platform, combined with offline debates in secondary schools, to investigate Belgian citizens’ opinions about the ethical, legal, and social implications (ELSI) of genomic information usage and, based on that, produce recommendations for health policymakers, experts and other stakeholders.

The DNA debate was disseminated as an open call through various communication channels to reach a large and diverse group of citizens among the Belgian population, but with specific attention to young generations since they will be the most impacted by the benefits and risks of genomic technologies (Council of Europe, 2019). Our communication strategy included newsletters from partnering organisations, national media (radio, television, and newspaper interviews), and social media (Facebook groups gathering teachers from secondary schools). Every Belgian citizen could voluntarily participate by creating an anonymous account on the dedicated website (Dutch version: dnadebat.be; French version: debatadn.be) by choosing their alias. It was possible to verify through Google Analytics that each contributor corresponded to a separate visitor on the platform. Before creating an account, participants were informed of the context and objectives of the debate, the rules of the platform, privacy statements, and how their contributions would be used.

Based on the Bpart tool creating platforms for online deliberation (Bpart, 2022), we provided participants with an informative and interactive environment stimulating their reflection, critical mind, and aptitude to produce well-argued-and-justified opinions. The platform was structured following the general format of participative intervention around mini-publics—inform, deliberate, produce—but within the limits of online environments (Felicetti, 2014; Parkinson and Mansbridge, 2012; Monnoyer-Smith and Wojcik, 2012). Traditional criteria to measure deliberation in offline contexts are, among others, interactivity, rationality of arguments and justification, equality in speaking opportunities, balancing personal experience and interests with the common good, mutual respect, authentic participation, and constructiveness (Hiratsuka et al., 2020; Friess and Eilders, 2015; Monnoyer-Smith and Wojcik, 2012). Some of these criteria can smoothly be transferred to the online context, such as equal opportunity in contributing, justification, rationality, and balancing personal interests with the common good. Yet some are harder to guarantee, such as the interactivity inside a large group of participants (Monnoyer-Smith and Wojcik, 2012). Besides, the group size and heterogeneity, response rate, topics discussed, and participants navigating through the platform strategically according to their specific needs are factors that do influence the degree of deliberation but cannot be controlled through the design and moderation of the online platform (Friess and Eilders, 2015). Online deliberation should learn from the quality criteria of more traditional forms. Nevertheless, one should not reduce it to them since its value lies in the new opportunities it offers.

The platform was designed around the informative and educational materials. Before contributing, every participant was confronted with different sources of information. They were first invited to watch a short video illustrating how genomics might intervene throughout the lifetime of every individual. They could then do an interactive test with 15 ethical dilemmas indicating what society would look like if everyone thought as them about genomics. The test results showed nine types of society participants could live in, illustrated by concrete situations worldwide involving genomic technologies. Finally, they were strongly encouraged to consult the educational package developed for secondary schools, additional literature, and nine case studies explaining how genomic technologies raise new ELSI in various contexts (King Baudouin Foundation, Sciensano, 2018). All these materials tended to be neutral by balancing risks and benefits and articulating competing perspectives and values at the individual, familial, and societal levels.

After being well-informed, participants could post their opinion about up to five open and challenging questions encouraging them to express their values, needs, concerns, and interests on how genomic data should be used now and in the future, independently of concrete applications and technical issues. These questions were:

1. What encourages me to learn more about my DNA, or what dissuades me from doing it? Why?
2. What motivates me to share my DNA data, or what stops me from doing so? Why?
3. A genetic passport for all: a good idea or not? Why?
4. How would DNA be used in my ideal society? What applications should be avoided? Why?
5. Do you want to share another idea about how we should deal with DNA in society?

A short description followed each question to contextualise it in the broader debate on genomic information usage. Participants could also vote, comment, and react to other participants’ contributions. Each contribution could only appear online after being moderated by the organising team to avoid offensive language and trolling. Yet, we limited that moderation to its strict minimum to allow all opinions to be heard.

Alongside the online platform, about 75 secondary schools all over Belgium responded to the open call to organise offline debates of about two hours in their classrooms. Schools proved to be the safest context to engage young generations (Council of Europe, 2019). We targeted students in the fifth and sixth grades (16–18 years old) because they were mature and skilled enough to debate subjects as complex as ELSI in genomics if supported by an educational package and supervised by their teacher. The educational package gathered the nine case studies, links to the online platform and its interactive tools, and deliberative exercises to help teachers challenge their students and moderate the debate. To validate their participation, students had to post their main conclusions, reflection, and opinion that emerged from the offline discussions on the platform. They could contribute individually, in sub-groups, or as a class.

In total, 4545 citizens interacted with the DNA debate website, from which 2581 filled in the interactive test, and 1127 voiced 1258 opinions under the five questions of the platform.

The present manuscript builds on the results from the general qualitative analysis of the 1258 contributions posted by the 1127 citizens on the online platform, which have been reported in a previous article (Mayeur et al., 2021). That first article provided an overview of the central norms and values surrounding genomic information usage among participants. These results came from an inductive thematic analysis using the NVivo 12 software. The themes emerged from the data without a conceptual framework that determined the initial coding, except for the research question determining the scope of the analysis (Braun and Clarke, 2006). The research question that guided this first analysis was the following: “How should genomic information be used on a societal level?” All five questions from the online platform were relevant to this research question. Hence, we considered all the contributions from citizens as one big narrative for this previous article.

For the present article, we revisited the 244 contributions of citizens related to the question of a genomic passport and its related codes when citizens responded to other questions from the platform (all data are publicly available in the Harvard Dataverse repository). We introduced the genomic passport to participants as genomic sequencing done on every citizen, which could be used throughout life within the healthcare setting for preventive, diagnostic, and therapeutic purposes, but outside as well. It illustrated the growing idea of personalised medicine and the increased collection of genomic data, eventually combined with other health data. Again, we performed an inductive thematic analysis of citizens’ contributions, but this time to answer the following research question: “What are the values and principles held by citizens on the potential implementation of a genomic passport?” This reanalysis of the data aimed to highlight how citizens consider its usage on a societal level and the ethical issues it raises. As we used an inductive approach, we started an open coding process, staying as close as possible to the contributions of citizens to let themes emerge naturally. That is how vulnerability emerged as a central and underlying theme that explains many other concepts expressed by the citizens, such as uncertainty, distrust, fears, and lack of security. These concepts represent the many faces of vulnerability in the contributions of citizens.

The Discussion section aims to put light on vulnerability as a latent and shared experience in the context of genomics because this particular type of vulnerability has not received the attention it deserves within the literature, especially not from the perspective of citizens. Researchers in ethics have thoroughly engaged with the ethical concerns surrounding patient groups who are particularly vulnerable (Gathron, 2019; McCormack et al., 2016). However, this conception of vulnerability did not map well onto our analysis since the type of vulnerability we encountered is not linked to groups of citizens who are a priori characterised as particularly vulnerable. Florencia Luna developed a unique understanding of vulnerability as overlapping layers of ontological and situational vulnerabilities (Luna, 2019) that best mirror how citizens from the DNA debate experience and perceive vulnerability in genomics. Building on the qualitative analysis of citizens’ contributions and the conceptual framework of Florencia Luna, this paper ends with some recommendations to mitigate the vulnerabilities of citizens within the context of a genomic passport for all.

## Results

### Participants’ understanding of the genomic passport

There are many interpretations of what a genomic passport would look like, how it would function, who would have access to which information, and the like. Mission creep is a concern here. Although a genomic passport may be introduced for purpose A, it may be used for other purposes in the future. Rather than limiting ourselves to one specific use of the genomic passport, we chose to keep its definition as open as possible to gain as much valuable input from citizens as possible about its various potential implementations.

Participants generally interpreted it as an official electronic record, either gathering only available genomic information or medical and health information of the individual as well. It would be generated in adulthood or at birth. In the latter case, one could interpret it as a broad newborn screening, an option currently being explored in the UK (Hopkins et al., 2021).

The inductive thematic data analysis took the influence of how participants interpreted the genomic passport on their opinions into account. For instance, some were against its creation because they thought it would be easily accessible, like a travel passport (value = privacy), while they only supported genomic information usage for medical purposes (motivation = improving health). Even if this interpretation of what a genomic passport might be is not realistic, the values, concerns, and motivations citizens voiced in this regard are still of great interest to any genomic data user.

The data analysis resulted in three categories of benefits of the genomic passport identified by participants, and three categories of drawbacks. One should not interpret these categories as exhaustive, yet they provide an overview of participants’ fundamental values, concerns, and motivations regarding a genomic passport for all.

### Benefits of a genomic passport

#### Advances in precision medicine and scientific research

According to participants, a genomic passport for all citizens would provide a global understanding of each individual’s and the overall population’s health, increase the knowledge of diseases, and, consequently, improve diagnostics, treatments, and prevention. It was the most supported added value of the genomic passport, showing that participants encouraged genomic information used to help others.

In certain circumstances, such as an accident or sudden hospitalisation, it can be helpful to make a quick and correct diagnosis, which takes the patient’s medical history into account. Science can also use the data stored in a scientific database, which can be used for all kinds of research and for finding new medications. (Contribution n°1136)

I think it’s a good idea to have a global view of our health, but who can have access to our passports? Normally I would say, doctors. But anyway, if my health data can help other people, then my passport will be welcome. (Contribution n°602)

#### More efficient patient management

Healthcare providers would manage patients more efficiently since they would have access to all relevant health, medical and genomic information.

The idea of a genomic passport seems to me to be extremely interesting for care and research. Indeed, it would allow for the evolution of treatments, discoveries, etc. If linked to our global medical file, it would allow us to be treated in a personalised and more efficient way, no matter where we are and what happens. I am thinking, for example, of someone who has a severe infection and eventually falls into a coma. We could easily see what he is allergic to, what diseases he has, what treatments he has already received, etc. That would make it much easier for the patient to be treated. I think it would make the doctors’ work much easier. (Contribution no. 971)

Some also argued that updated genomic passports could avoid abuse of medical examination and prescription of inappropriate treatment, thereby reducing private and public expenditures.

Beyond a genomic passport, it would be USEFUL that each citizen has a HEALTH REPORT IMPERATIVELY completed during ALL health interventions (consultations, diagnoses, treatments). It is IMPERATIVE to limit for some patients, doctors, and hospitals the abuse of prescriptions and examinations in the name of the “profitability” required by hospital infrastructures. That would keep the possibility of a Social Security system, which puts all citizens in a relationship of solidarity. (Contribution no. 24)

Finally, some claimed the right to freely choose to know (or not) the information contained in their genomic passport after being informed about the benefits and harms.

I do think that every citizen can choose what happens with their passport. Do they want to know what is in it? Do they just want to have it already so that new healing can be worked on, and when they need it, these healings are already there? Do they just want to know a part of it? It is all their own choice. (Contribution no. 737)

Indeed, some participants were afraid of knowing their genomic information because they thought that they would know everything or nearly everything about their past (e.g., genealogy), present (e.g., diagnosed diseases, character, talents), and future (e.g., predispositions and even their death).

In life, there is a beginning and an end to everything. Why bother to know everything about our health and to treat everything? Wanting to know what is likely to happen is a bad idea. You must live, and will happen what will happen. That’s it. Dying and having diseases are part of life. Knowing when you are likely to die or develop a severe illness will block many people. We will live in an anxious world where most people will no longer be able to live normally. You have to live, do the things you want to do, and the fate that awaits you will come. You must live, do the things you want, and the fate awaiting us will happen (Contribution no. 100)

I think it is really unnecessary to know everything about yourself. You push people into pigeonholes. You tell people how they should be instead of letting themselves discover it, something more humane. One could say, for example, that your talents lie in languages, yet you would like nothing better than to study something in the field of science. Could schools exclude you from the scientific option because they do not see any chance of success in you? You might also start to doubt yourself and never even dare to do anything with science. Wouldn’t that be a shame? Even for medical purposes, I don’t see the point. If you know that a family member has a genetic disorder, you can just go to the annual check-up. It is not necessary to scare people before they have the condition itself. Say they already have the disorder, then some genes might never be expressed, even if the person is a carrier. So why all that fear in advance? (Contribution no. 1153)

#### Genomic information usage for forensic purposes

Although a few strongly opposed this, most participants expected the genomic passport usage for forensic purposes to facilitate the search for criminals, maybe dissuade potential offenders from acting, and, consequently, improve national security.

I think a genomic passport is quite a good idea when it falls into the hands of the right people. It should only be used for police investigations, for example. In this way, offenders can be tracked down much faster and be a danger to society for less time. (Contribution no. 1152)

### Drawbacks of a genomic passport

#### Arguments based on a sense of vulnerability due to distrust and fear of losing control

Some participants argued that governments and private companies already know too much about individuals by collecting and storing large amounts of personal data daily. They worried that a genomic passport for all would exacerbate the control over the population and tracing of individuals, rendering those individuals and their relatives vulnerable and diminishing their liberty and agency (e.g., putting pressure on individuals to take their genomic information into account).

I do not think it is a good idea to make genomic passports for the population. It is no longer about cameras or fingerprints that can provide more safety. It’s really about data that gives you information about your physical characteristics, personality, ethnic origin and much more. You put yourself and your close and even distant family members into a vulnerable position when the government has insight into all your data. (Contribution no. 541)

Additionally, centralising genomic information within a genomic passport increases the risk that actors outside healthcare, including insurers, employers, educational bodies, banks, or any commercial company, could use this information to categorise, discriminate or exclude people based on genetic makeup or predispositions to incurable diseases. A few also feared discrimination inside the healthcare system (e.g., limited access to care and reimbursement). In other words, participants deemed it unacceptable that their genomic passport could negatively change the way people consider and treat them.

You classify people literally according to their health. […] Society, amongst others, healthy or better-off people, will despise people with severe diseases or incurable illnesses, which causes a conflict between each other. You can break relationships with this (with family or friends). Because people always prefer healthy people. […] And they cannot do anything about it. (Contribution no. 747)

#### Arguments based on the feeling of lack of security

Participants’ sense of vulnerability and fear of abuse (e.g., invasion of privacy and discrimination) generated a feeling of unease. Participants had doubts about existing security systems and a sufficiently robust legal framework to protect them against the many potential misuses in various fields. Losing their privacy and control over their most personal information made them anxious.

Personally, it seems that too much information on these DNA passports opens up many ways to abuse them. It seems complicated to keep this information secret, and companies will always make profits from such information. Just the idea that all the information about me is stored somewhere (even things I might not know myself) gives me a suffocating feeling. (Contribution no. 1054)

Participants expressed the need to keep control of their genomic passport, including transparency and autonomy in its uses, such as knowing and deciding who has access and for which purposes.

I think that citizens should have the right to know precisely what is done with which information because it is personal information (and maybe also sensitive). (Contribution n°388)

The right to decide who may use the data must lie with each individual. (Contribution no. 29)

Some argued that only qualified users, bound by professional secrecy and having a special request in a specific context, should be able to use the genomic passport. Furthermore, it should only include limited and relevant health and genomic information (e.g., severe diseases) to reduce the risk of misuse.

The problem is that your passport might be exchanged very quickly. It is still your choice who you share it with and who you do not. Doctors, therefore, have professional secrecy and are not allowed to do anything with the DNA unless you indicate otherwise. (Contribution no. 894)

#### Arguments based on uncertainty

Participants’ wish to keep control over their genomic passport can also be explained by the fundamental uncertainty about the current and future uses of genomic information. That uncertainty made participants anxious since they considered this information most personal and intimate.

The biggest problem is that you are vulnerable. And not only you but your family too. You give away genomic information from you and your family. Who can handle it all? What will they do with it? Just having to live in that uncertainty should not be allowed. (Contribution no. 866)

## Discussion

Our results give the concept of vulnerability a central role in understanding participants’ values, motivations, and concerns regarding a genomic passport. Participants’ feelings of vulnerability may have diverse but interrelated explanations.

Many participants were worried about losing control of such intimate information, especially since its current and future uses are uncertain. Data misuse would not only negatively impact the lives of individuals who share their data, but their close and distant relatives also run the risk of being affected, participants pointed out, meaning that their feeling of vulnerability exceeds their person. Participants’ awareness of eventually exposing their relatives and descendants at risk when sharing their genomic data has been reported many times (Dheensa et al., 2019; Middleton et al., 2019; Milne et al., 2019; Castell et al., 2019; Mayeur and Van Hoof, 2021; Haeusermann et al., 2018; Comité Consultatif national d’éthique, 2018; Middleton et al., 2016). The perspective of gathering so much sensitive information in a genomic passport or any other type of health record generated a feeling of vulnerability among participants. Privacy protection in genomics is particularly complex, given that anonymising data does not guarantee they will never be re-identifiable (Grebe et al., 2020). In a previous study, participants interviewed about their experience and perception of privacy in direct-to-consumer genetic testing viewed the risk of losing control of their data as inherent to a more realistic conception of privacy, testifying their mistrust in security measures to protect them from abuses (Haeusermann et al., 2018).

Participants of the DNA debate identified many potential misuses in various fields. Exclusion and discrimination by insurers, employers, commercial companies, and the government were the most cited examples, which aligns with the results from previous public engagement initiatives (Hopkins, Kinsella and Evans, 2021; Rivas Velarde et al., 2021; Mayeur and Van Hoof, 2021; Dheensa et al., 2019; Middleton et al., 2019; Haeusermann et al., 2018; Middleton et al., 2016). In addition, forensic uses of the genomic passport could lead to extensive profiling where people with specific genetic features or predispositions, such as aggressiveness, would become suspicious by default. The genomic passport may thus render individuals vulnerable because it increases the power of people who could use this information against them. The general distrust of participants in potential users of their genomic data highlights the significance of these users understanding and taking the citizens’ vulnerability into account and setting up appropriate measures to mitigate this vulnerability and protect citizens against abuse. The conceptualisation of trust by the philosopher Katherine Hawley might be helpful in this context. Trust implies that an individual (A) agrees to show vulnerability to an entity/individual (B) by entrusting the latter with something A cares about (e.g., genomic information). As a consequence, A expects B not to abuse its power to harm A through an explicit (e.g., consent) or implicit (e.g., fulfilling its role of protection) commitment (Hawley, 2019). Distrust can thus be defined as a rational attitude (e.g., analysing the risks of a given situation) or primary instinct (e.g., an intuition resulting from the subconscious analysing the risks) whose role is to protect individuals from entrusting their vulnerability to people likely to abuse it. As previously described by Onora O’Neill, we should aim for well-placed trust, not a blind leap of faith (O’Neill, 2004). In line with this idea, the majority of participants from two independent citizen fora prioritised harm prevention by imposing sharing restrictions for driven-profit actors over individual freedom (Rivas Velarde et al., 2021; Mayeur and Van Hoof, 2021).

The last reason that may explain why participants of the DNA debate felt vulnerable only concerns a minority. A few participants thought that sequencing their genome to create a genomic passport equals knowing and understanding everything about their genome, at least to the extent of current scientific knowledge. These participants seem to confuse genomic sequencing and interpretation of results, even if it is true that sequencing the genome aims at understanding it as much as possible. Likewise, a few contributions from the DNA debate depict a more deterministic conception of genomics. If genome sequencing reveals information about the past (e.g., genealogy), present (e.g., diagnosed diseases, character) and future (e.g., predispositions) of individuals, the general public sometimes lacks education on the limits and practical utility of genomic information. Those deterministic conceptions among the general public have already been underlined in previous studies (Carver et al., 2017; Wong et al., 2004). Yet, beyond determinism, these contributions of participants mainly reveal a strong link between their genome and their identity, justifying their feeling of vulnerability. Public engagement initiatives have often echoed the close connection between identity and genomic information (Hopkins et al., 2021; Ballard et al., 2020; Panofsky and Donovan, 2019; Shim et al., 2018).

In particular, the metaphor of the layers of vulnerabilities is relevant to describing the complex interactions between the different types of vulnerabilities expressed by participants regarding the idea of a genomic passport for all. This metaphor, developed by Florencia Luna, illustrates that all humans are vulnerable, but to various degrees, depending on whether external factors do or do not activate extra layers of vulnerability (Luna, 2019).

The first layer of vulnerability participants of the DNA debate referred to is ontological vulnerability. That concept relates to the fact that all humans are unavoidably vulnerable because of their mortality and exposure to harm, whether physical (e.g., diseases), psychological (e.g., anxiety), or others (Luna, 2019; Morais and Monteiro, 2017; Delgado Rodriguez, 2017; Rogers et al., 2012). Participants seem to view DNA as a personal code embodying their ontological vulnerability because it informs them about their constitutional weaknesses (e.g., low tolerance to stress) and their predispositions to or current diseases. In other words, establishing a genomic passport for all citizens confronts them with the harsh reality that everyone is at risk of health problems, even the youngest and healthiest persons.

The second layer of vulnerability participants expressed is situational vulnerability, which is the risk of being harmed by external factors, whether social, environmental, cultural, political, or economic (Luna, 2019; Morais and Monteiro, 2017; Delgado Rodriguez, 2017; Rogers et al., 2012). Some actors could use the genomic passport for their interests at the cost of individuals, while citizens expected its usage in their interests (e.g., health) and the common good (e.g., public health and scientific research). Exploitation, discrimination, inequalities, exclusion, and stigmatisation based on one’s genetic makeup were examples frequently cited. Insurers or employers could discriminate or exclude their clients/employees at higher risk of rare or incurable diseases, causing psychological, social, and economic harm.

The ontological layer of vulnerability cannot be eliminated since it is inherent to human beings. However, genomic data users have an opportunity to mitigate the health threats inherent to ontological vulnerability by using the genomic passport for prevention and timely diagnosis and treatment. If this passport would show citizens their vulnerabilities related to health, it could alleviate many, provided it aims to counter harmful predispositions and diseases. Nevertheless, the genomic passport can also create clinical vulnerability, as conceptualised by Sossauer, Schindler and Hurst, namely a mismatch between the claim of the patient and the service provided by the healthcare professionals (Sossauer et al., 2019). According to these authors, this type of vulnerability is influenced by the patient’s characteristics (e.g., personality or socio-economic situation) and external factors (e.g., the cost of treatment). For instance, the genomic passport might reveal a predisposition to a rare genetic disease (patient characteristic) whose treatment costs or preventive measures are too high (external factors) to be covered by the individual (personal economic situation) or reimbursed by the healthcare system (societal organisation). The genomic passport would put these patients in a distressing situation of dependence and clinical vulnerability because of a mismatch between the high diagnostic potential of the healthcare system and its more limited preventive and curative potential. Two-tier healthcare that reinforces inequalities worried participants of the DNA debate when reflecting on the eventuality of a genomic passport for all.

All genomic data users play a role in alleviating situational vulnerability. They must identify external factors activating those layers to erase them where possible or avoid or limit them (Luna, 2019). While one cannot erase ontological vulnerability, one can merely mitigate it by adding extra layers of protection against situational vulnerabilities. The literature in bioethics often proposes protective and preventive measures as strategies to alleviate situational vulnerability (Luna, 2019; Morais and Monteiro, 2017; Rogers et al., 2012). Regarding the genomic passport, it means ensuring optimal data security to prevent a breach of privacy, legally prohibiting non-legitimate actors from accessing those data, such as insurers and employers, and providing for sanctions in the case of infringement. Citizens interviewed about privacy in direct-to-consumer genetic testing stressed the role of governments in eliminating social and health inequalities that expose some to more discrimination and privacy issues. Ensuring the equal distribution of the right to privacy among citizens should be a priority, according to these participants (Haeusermann et al., 2018).

But solely relying on protective and preventive measures may lead to paternalistic attitudes if not combined with measures that aim to empower individuals (Luna, 2019; Morais and Monteiro, 2017; Delgado Rodriguez, 2017; Rogers et al., 2012). Genomic data users bear the responsibility of respecting individuals as autonomous agents who are more than their genetic makeup. Moving beyond genetic determinism is a challenge also stressed by participants from the Genomics England initiative (Hopkins et al., 2021), which can empower individuals in their relationship with their genomic information. Some participants of the DNA debate feared the genomic passport would pressure them to become acquainted with its information and act upon it. They argued that knowing what might happen to their and their relatives’ health could generate psychological harm, affect family relationships, induce a false sense of control over life and eliminate its carefreeness. Some participants from the Genomics England initiative shared the same concerns and warned against generalised whole-genome sequencing in newborns leading to a risk-averse society that would “sterilise life to a certain extent” to borrow the words of a participant (Hopkins et al., 2021, p. 39). Participants from their and our initiative claimed that one should respect individual choices and preferences about knowing and considering genomic information as they vary highly from one individual to another and may significantly impact their life (Hopkins et al., 2021). For instance, individuals suffering from their disease and all its negative consequences might feel particularly anxious and vulnerable about knowing their relatives’ risks to diseases and informing them about these (Kerr et al., 2018). Biopolitics, supported by the genomic passport, should not foster paternalistic attitudes through a zero-risk health imperative, whether the risks be behavioural or constitutional (e.g., genetic predisposition) (Morais and Monteiro, 2017).

If both ontological and situational vulnerabilities generally apply to all domains of life, the fundamental uncertainty surrounding genomics exacerbates it even more. First, genome sequencing oftentimes informs about probabilities and risks, which are difficult to interpret, whereas these may have a decisive impact on individuals. Participants from the Genomics England initiative pointed out the need to educate the public in dealing with and rationalising this first degree of uncertainty (Hopkins, Kinsella and Evans, 2021). The widespread implementation of genomic passports generates a need for high-quality counselling to assist people in handling such personal and potentially high-impact knowledge to limit its potential damage (Grebe et al., 2020). Second, individuals whose genome is sequenced cannot predict additional results discovered by scientific advances. Third, genomics is evolving rapidly without knowing its limits and possibilities, like any new science (Government Office for Science, 2022). When storing or sharing genomic data, no one can be sure about their future uses, which means there is a constant threat of misuse and abuse.

In other words, even when all measures have been taken to avoid, reduce or eradicate situational layers of vulnerability in genomics, there is always the risk that unpredictable factors activate the latent ontological vulnerability embodied by our genome. This perennial vulnerability represents a constant threat of harm to individuals, which actors using genomic information, including citizens, should always keep in mind even when they have the best intentions. That should improve the reliability and responsibility of actors using genomic information—and maybe the genomic passport in the future—strengthening, in turn, citizens’ trust in genomics. The recent suggestion by McMahon et al. to install ‘Harm Mitigation Bodies’ that people could turn to when they feel they have been harmed by the misuse of their health data is an example of a possible initiative that can encourage collective responsibility and inspire more trustworthy use of genomic data (McMahon et al., 2020). Improving and nurturing citizens’ trust regarding genomic information usage could be the starting point in alleviating their sense of vulnerability. In this sense, trust can be interpreted as a wager on something or someone after analysing its risks and benefits while knowing that the full extent of these risks and benefits is unknown (Braun et al., 2021). This understanding of trust brings its fragility to light, especially in genomics, where uncertainty is ubiquitous. One should never forget that citizens’ trust in genomics is as fragile as the apparent silence of their ontological vulnerability. Since it can be disturbed at any time, it requires constant attention.

Finally, one way to ensure a better match between the goal of genomic data users and the expectations and fears of citizens is to conduct public engagement initiatives supported by policymakers and experts in genomics. Public engagement empowers citizens as relevant stakeholders whose concerns, values, and interests must be understood and considered in the public health policy agenda and daily medical practice. Since citizens have their own experience of vulnerability in genomics, genomic data users should listen to them and understand it to address it most effectively and appropriately. While public engagement is most valued because it improves citizens’ trust, one should not dismiss the positive function of distrust. Their mistrust in technologies—such as genomics—steers citizens to prudence towards its risks, frame its power, control its evolution, and examine the interests at stake and the objectives to meet these interests (Braun et al., 2021).

### Limitations

Our method presents several limitations. First, citizens who contributed to the DNA debate were not representative of the Belgian population. Our goal was to gather diverse contributions from a large group of citizens, with specific attention on young generations, after informing them neutrally, to provide a rich qualitative analysis of the values one can find within citizens’ opinions. Second, the DNA debate is not a scientific study per se and does not pretend to be. It is a vast online public engagement initiative whose results have been analysed using an inductive thematic approach. Third, this article does not offer a taxonomy or classification of the concept of vulnerability in genomics in the view of citizens since further empirical research involving the general public is needed to complete, confirm or question our results. Particularly in other countries, it may be the case that other factors, such as social, cultural, or economic ones, influence how citizens experience vulnerabilities in genomics.

## Conclusion

Confronted with the idea of a genomic passport for all citizens, participants from the Belgian online DNA debate expressed a sense of vulnerability in two ways. First, in their view, the genomic passport is a personal code embodying their ontological vulnerability. It reminds them that they are unavoidably vulnerable due to constitutional factors, such as psychological frailty (e.g., anxiety), physical weakness (e.g., diseases), and finitude (death). Second, genomic data misuse can exacerbate this first perennial vulnerability by adding situational vulnerabilities, such as exploitation, discrimination, inequalities, exclusion, and stigmatisation based on genetic makeup.

We propose some policy recommendations to alleviate these vulnerabilities inherently linked to genomic technologies usage in a public setting. These recommendations combine individuals’ empowerment, protective measures, and trust:

- Any implementation of a genomic passport should aim at improving preventive and curative health care provision for the citizens whose genomic information is catalogued to avoid genetic vulnerabilities (e.g., predispositions) becoming concrete harms (e.g., diseases). However, public health policies should not fall into a health imperative imposing paternalistic measures, for instance, pressuring individuals to know and take their genomic information into account. Adequate information, education, and respect for autonomy could empower individuals dealing with their genomic information throughout life.
- All genomic data users should also identify external factors causing situational vulnerability to erase, avoid or minimise them. They should ensure that protective and preventive measures are implemented—such as data security, prohibiting access to non-legitimate actors, and legal sanctions in the case of infringements.
- Organising public engagement initiatives supported by policymakers and genomic experts have two main advantages. First, they ensure that citizens’ values and concerns, such as vulnerability, are understood and considered in the public health policy agenda and daily medical practice. Second, they could improve citizens’ trust in genomics and thus relieve part of their sense of vulnerability.

## Data Availability

All data generated and analysed during the DNA debate, namely all the contributions posted by citizens on the online platform, are available in the Harvard Dataverse repository: 10.7910/DVN/TT00UU.
